# Bayesian estimation of cell type–specific gene expression with prior derived from single-cell data

**DOI:** 10.1101/gr.268722.120

**Published:** 2021-10

**Authors:** Jiebiao Wang, Kathryn Roeder, Bernie Devlin

**Affiliations:** 1 Department of Biostatistics, University of Pittsburgh, Pittsburgh, Pennsylvania 15261, USA;; 2 Department of Statistics and Data Science, Carnegie Mellon University, Pittsburgh, Pennsylvania 15213, USA;; 3 Computational Biology Department, Carnegie Mellon University, Pittsburgh, Pennsylvania 15213, USA;; 4 Department of Psychiatry, University of Pittsburgh School of Medicine, Pittsburgh, Pennsylvania 15213, USA

## Abstract

When assessed over a large number of samples, bulk RNA sequencing provides reliable data for gene expression at the tissue level. Single-cell RNA sequencing (scRNA-seq) deepens those analyses by evaluating gene expression at the cellular level. Both data types lend insights into disease etiology. With current technologies, scRNA-seq data are known to be noisy. Constrained by costs, scRNA-seq data are typically generated from a relatively small number of subjects, which limits their utility for some analyses, such as identification of gene expression quantitative trait loci (eQTLs). To address these issues while maintaining the unique advantages of each data type, we develop a Bayesian method (bMIND) to integrate bulk and scRNA-seq data. With a prior derived from scRNA-seq data, we propose to estimate sample-level cell type–specific (CTS) expression from bulk expression data. The CTS expression enables large-scale sample-level downstream analyses, such as detection of CTS differentially expressed genes (DEGs) and eQTLs. Through simulations, we show that bMIND improves the accuracy of sample-level CTS expression estimates and increases the power to discover CTS DEGs when compared to existing methods. To further our understanding of two complex phenotypes, autism spectrum disorder and Alzheimer's disease, we apply bMIND to gene expression data of relevant brain tissue to identify CTS DEGs. Our results complement findings for CTS DEGs obtained from snRNA-seq studies, replicating certain DEGs in specific cell types while nominating other novel genes for those cell types. Finally, we calculate CTS eQTLs for 11 brain regions by analyzing Genotype-Tissue Expression Project data, creating a new resource for biological insights.

Gene expression quantified at the tissue level, bulk gene expression data, has been a useful resource for understanding the etiology of different diseases. RNA sequencing technology, applied to tissue samples, is mature, and its relatively cost-efficient property allows assessment of tissue from hundreds of samples, thereby producing rich data sets (Allen et al. 2016; Parikshak et al. 2016; The GTEx Consortium 2017; Bennett et al. 2018; Wang et al. 2018). However, because tissue is comprised of a variety of cell types, bulk data are the convolution of gene expression from myriad cells of various cell types. To overcome this challenge, researchers have pursued single-cell RNA sequencing (scRNA-seq) to quantify cell type–specific (CTS) gene expression, either at the cellular or nuclear level (Darmanis et al. 2015; Mathys et al. 2019; Velmeshev et al. 2019). While providing important insights into etiology, such data have their own limitations: cells are typically collected from a limited number of samples, thus they lack sufficient variation over samples; and the data are noisy and technically variable owing to quantification of a small number of RNA molecules. This issue is especially severe for single-nucleus RNA-seq (snRNA-seq) data from frozen tissue, which is the main specimen source for brain research. Nuclear RNA accounts for only 20%–50% of the RNA molecules in the whole cell, and this fraction varies across cell types (Bakken et al. 2018). Furthermore, studies of brain tissue have found that snRNA-seq fails to detect a fraction of the microglia population (Mathys et al. 2019) and microglial activation in the human brain (Thrupp et al. 2020), yet microglia are thought to be a key cell type related to critical diseases, such as Alzheimer's disease.

To overcome the drawbacks of bulk and scRNA-seq/snRNA-seq data while maintaining their unique advantages, we propose to integrate bulk and single-cell data to estimate CTS expression for large samples. Existing methods typically can only estimate population-average CTS expression (e.g., csSAM) (Shen-Orr et al. 2010). To enable subject-level estimation, we previously developed a novel MIND algorithm (Wang et al. 2020) that extends population-average estimates to the level of subject and cell type by borrowing information across multiple measures of bulk level expression from the same subjects. We implemented the MIND algorithm within the framework of mixed-effects models and estimated the subject-level CTS expression via empirical Bayes. Although MIND's estimates of CTS expression are useful in subject-level analyses, multimeasure bulk expression data are not commonly available. Instead, most data sets only have one or two measures of bulk expression per subject. Correspondingly, there have been methods developed in parallel for single-measure bulk DNA methylation data (e.g., TCA) (Rahmani et al. 2019) and gene expression data (e.g., CIBERSORTx) (Newman et al. 2019). TCA is a frequentist method similar to MIND, and CIBERSORTx relies on non-negative least squares to estimate sample-level CTS expression with the goal of separating two groups of samples. There are also CTS analytical methods for testing the interaction of cell type fractions and the variable of interest without explicit estimation of CTS expression, such as CellDMC (Zheng et al. 2018), which was originally designed for DNA methylation data. Nonetheless, these methods have not efficiently used the rich information available from single-cell data.

To address these deficiencies, we develop a Bayesian MIND (bMIND) algorithm to refine the estimation of CTS expression for each bulk sample. As compared to MIND, bMIND not only works for bulk data without multiple measures, but it can also be used to estimate the sample-level CTS expression for each brain region, for instance, thereby enabling a study of heterogeneous CTS expression patterns across brain regions. To provide accurate and reliable estimates in this setting, we propose to use information from scRNA-seq data by incorporating it as prior in a Bayesian analysis. Specifically, we extract informative prior distributions of mean CTS expression and covariance structure for each gene from scRNA-seq data to facilitate the estimation of sample-level CTS expression from bulk data. We adopt a Bayesian approach because it is known to work well and robustly by incorporating prior information to regularize the statistically challenging estimation we aim to achieve in this work. Distinguishing itself from other methods, bMIND is a powerful and flexible tool, suitable for estimation of CTS expression and for testing for differential expression. The approach works best when scRNA-seq data are available, but it also works well without prior information.

Here, we introduce the bMIND algorithm and compare it to other state-of-the-art methods. We also show its utility by various analyses, including CTS differential expression analysis of data relevant for autism spectrum disorder (ASD) and Alzheimer's disease (AD). Moreover, analyzing updated Genotype-Tissue Expression Project (GTEx) V8 brain data, we calculate CTS eQTLs for each of 11 brain regions to create a new resource for uncovering the etiologies of complex diseases and other phenotypes.

## Results

### Bayesian estimation of sample-level CTS gene expression

**Figure 1.**
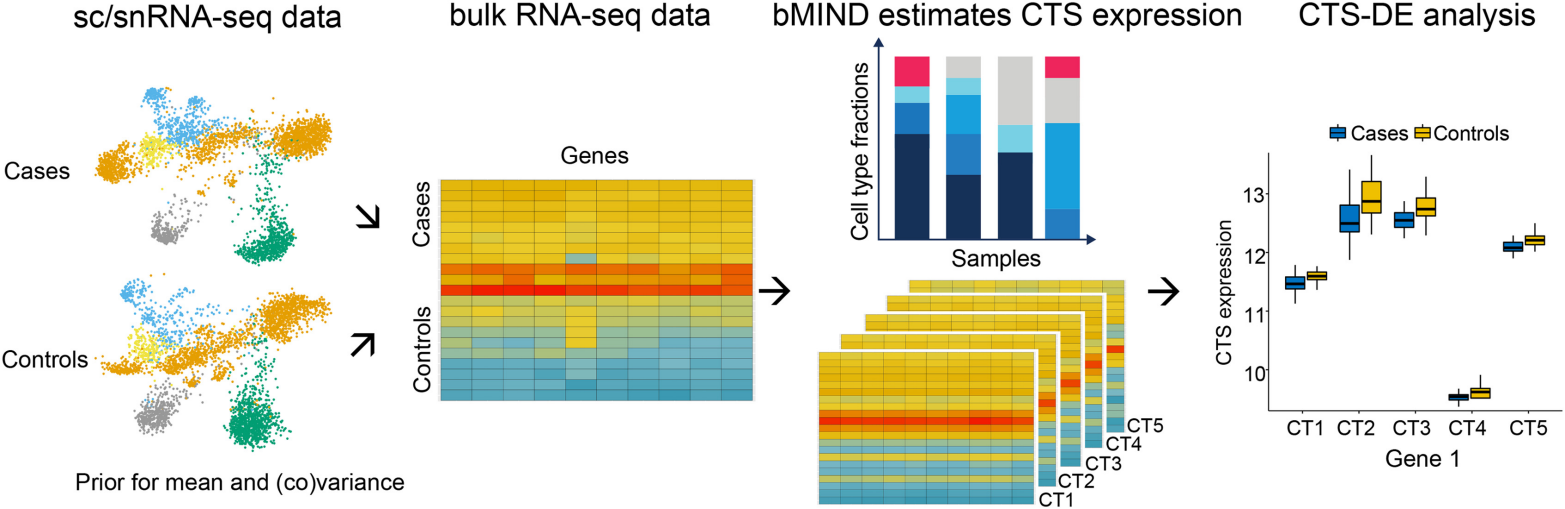
Overview of bMIND algorithm and CTS differential expression analysis (CTS DE). With prior information from scRNA/snRNA-seq data for case and control subjects, bMIND analyzes bulk RNA-seq data and estimates sample-level CTS expression with a Bayesian approach. Here, we present an example of five cell types (CT1–CT5). In the downstream analysis, as an example, we test the association between CTS expression and phenotype for each gene in each cell type and identify CTS differentially expressed genes (CTS DEGs).

To improve the estimation of sample-level CTS expression, we propose a Bayesian algorithm (bMIND) to incorporate prior information from single-cell data (Fig. 1). We model bulk expression of sample *i* in gene *j*, ***x***_*ij*_, for *T* ≥ 1 measures, as a product of cell type fraction (***W***_*i*_, *T* × *K*) and CTS expression (***a***_*ij*_, *K* × 1) of *K* cell types in Bayesian mixed-effects models

(1) $$\begin{array}{rl} x_{ij} & =W_{i}a_{ij}+c_{i}^{{\left(1\right)}^{\prime }}{\text{β}}_{j}+W_{i}B_{j}c_{i}^{\left(2\right)}+e_{ij},\\ a_{ij} & ∼N(\alpha_{j},\Sigma_{j}),e_{ij}∼N(0,\sigma_{j}^{2}I_{T}),\;(1) \end{array}$$

where ***α***_*j*_ (*K* × 1) is the expected CTS expression for the *j*th gene that constitutes the profile matrix, $\Sigma_{j}$ (K×K) is the covariance matrix of CTS expression for *K* cell types, $c^{\left(1\right)}$ denotes covariates affecting bulk expression, $c^{\left(2\right)}$ represents covariates affecting CTS expression, and $e_{ij}$ is the error term that captures the unexplained random noise with variance $\sigma_{j}^{2}$. The cell type fraction ($W_{i}$) is assumed known or preestimated using a cell type fraction estimation algorithm (Wang et al. 2019; Jew et al. 2020). The goal of bMIND is to provide the posterior mean of the CTS expression ($a_{ij},K\times 1$) for each sample *i*, gene *j*, and *K* cell types.

To incorporate information from scRNA-seq data, we use these summary statistics: for each gene *j*, let ${\hat{\alpha }}_{j}$ be the profile matrix, and let ${\hat{\Sigma }}_{j}$ be the cell type covariance matrix. We assume the following prior distribution

- $\alpha_{j}∼N({\hat{\alpha }}_{j},0.5I_{K}),$ where ${\hat{\alpha }}_{j}$ is the average CTS expression calculated from scRNA-seq data;
- $\Sigma_{j}∼\mathrm{Inv\_Wishart}({\hat{\Sigma }}_{j},50)$ where the first parameter is the expected covariance matrix and the second parameter represents the degree of belief; the inverse-Wishart distribution is the conjugate prior for the covariance matrix, which eases estimation, and facilitates explicitly incorporating the prior covariance matrix ${\hat{\Sigma }}_{j}$ or the *j*th gene estimated from scRNA-seq data; and
- $\sigma_{j}^{2}∼\mathrm{Inv\_Wishart}(1,0)$, which is non-informative.

Given the technical noise and variability of scRNA-seq data, we use summary statistics from the scRNA-seq data rather than the raw data because summary statistics are more robust and also reduce the computation burden (Zhu et al. 2018). The hyperparameters in the prior distributions are chosen based on empirical experiments. bMIND is robust to their specification, as shown in “Results.” We allow gene-specific parameters and analyze each gene in parallel. Although implemented with Markov chain Monte Carlo (MCMC) sampling, bMIND is computationally efficient. Depending on the sample size and number of cell types, all genes in the genome can be analyzed in approximately an hour using 30 CPU cores.

### CTS differential expression analysis

When the samples are from cases or controls, we include the case-control status (y) into the model for differential expression analysis. We use a Bayesian framework for testing $H_{0}:a_{j}^{0}=a_{j}^{1}$ for each cell type; where $a_{j}^{0}$ and $a_{j}^{1}$ denote the mean CTS expression for gene *j* in controls and cases, respectively. This new model allows the incorporation of prior information from cases and controls of single-cell studies:

(2) $$x_{ij}=I(y_{i}=0)W_{i}a_{j}^{0}+I(y_{i}=1)W_{i}a_{j}^{1}+c_{i}^{{\left(1\right)}^{\prime }}{\text{β}}_{j}+W_{i}B_{j}c_{i}^{\left(2\right)}+e_{ij},$$

where $x_{ij}$ represents bulk expression for the *i*th sample in the *j*th gene , *y*_*i*_ is the disease status with 0 for controls and 1 for cases, and $W_{i}$ denotes the cell type fractions. We use prior distributions $a_{j}^{0}∼N(\alpha_{j}^{0},\Sigma_{j}^{0})$ and $a_{j}^{1}∼N(\alpha_{j}^{1},\Sigma_{j}^{1})$ for cases and controls separately. With $a_{j}^{0}$ and $a_{j}^{1}$ as mean parameters for cases and controls, we generate their Bayesian posterior samples and calculate the MCMC *P*-values by comparing the posterior distribution of $a_{j}^{0}-a_{j}^{1}$ with the null (0). Alternatively, we can also perform CTS DE analysis with the estimated sample-level CTS expression from Equation 1 (see the specific testing procedure in Methods).

For comparison, we use four other methods: (1) TCA (Rahmani et al. 2019), a frequentist approach similar to MIND, designed for bulk DNA methylation data, but also applicable for CTS estimation of gene expression; (2) CIBERSORTx (Newman et al. 2019), which estimates CTS expression via non-negative least squares; (3) csSAM (Shen-Orr et al. 2010), designed for microarray data to estimate population-average CTS expression, and featuring a permutation-based test for CTS DE analysis; and (4) CellDMC (Zheng et al. 2018), designed for DNA methylation data, but applicable to gene expression data. CellDMC tests CTS DEGs by regressing bulk expression on the interaction terms between phenotype and cell type fractions, without estimating CTS expression. We also compare bMIND, which uses a prior derived from scRNA-seq data, with bMIND_rp (a variant of bMIND that uses a rough prior based on the analyzed bulk data) and bMIND_np which uses non-informative prior.

### bMIND refines estimates of sample-level CTS expression

**Figure 2.**
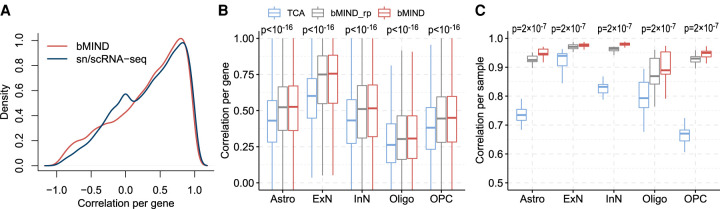
Correlation comparison for estimated sample-level CTS expression. (*A*) Correlation of average CTS gene expression (across samples), over cell type, obtained from two brain RNA-seq data sets (Parikshak et al. 2016; Velmeshev et al. 2019). For a benchmark, we assess the concordance between brain snRNA-seq data (Velmeshev et al. 2019) and scRNA-seq data (Darmanis et al. 2015) (labeled as sn/scRNA-seq). (*B*,*C*) Using realistic simulations, correlation between truth and estimated CTS expression are computed to compare TCA (Rahmani et al. 2019) with bMIND and bMIND_rp (using a rough prior). The task involved analyzing pseudo-bulk data generated from snRNA-seq data (Velmeshev et al. 2019) obtained from ASD subjects, and bMIND uses a prior derived from the corresponding controls. For each cell type, we compute correlation across samples for each gene (*B*), and correlation across genes for each sample (*C*). The *P*-values are obtained from paired one-tailed Wilcoxon test comparing TCA and bMIND_rp.

We evaluated the properties of bMIND with real-data analyses and realistic simulation studies. First, we checked if bMIND was able to detect variation in gene expression by cell type. We tested this by looking for consistent CTS expression across different data sets, using two independent bulk RNA-seq data sets from brain samples of subjects diagnosed with ASD and samples from unaffected subjects (Parikshak et al. 2016; Velmeshev et al. 2019) and independent priors derived from snRNA-seq data (Velmeshev et al. 2019) and scRNA-seq data (Darmanis et al. 2015). We paired Darmanis's scRNA-seq data, used as a prior, with bulk data from Parikshak et al. (2016); likewise, we paired Velmeshev's snRNA-seq data with bulk data from Velmeshev et al. (2019). To ensure the prior did not exert too much influence, we set the variance of the prior distribution of the expression profile matrix to 1000. After averaging the estimated CTS expression across samples within each data set for each gene, we calculated the correlation between the two averages over cell types. We performed the same comparison by computing the correlation of snRNA-seq (Velmeshev et al. 2019) and scRNA-seq estimated gene expression data (Darmanis et al. 2015). The correlation in the profile matrix estimated from two independent applications of bMIND was comparable to that from sc/snRNA-seq data sets (Fig. 2A). These results show that bMIND provides meaningful estimates of gene expression profiles derived from bulk data sets.

Next, we assessed whether bMIND could provide reliable sample-level CTS estimates. Ideally, this would be evaluated by comparing scRNA-seq and CTS estimates obtained from the same samples, but in a comparison of bulk RNA-seq and reconstructed bulk expression obtained from snRNA-seq data, the per gene correlation was observed to be quite low (Velmeshev et al. 2019). We instead used simulations to assess the correlation between estimated and true CTS expression for each cell type. Velmeshev et al. (2019) collected snRNA-seq data from brain tissue samples of subjects diagnosed with ASD and samples from unaffected subjects as controls. Using the single-nucleus expression for the available 41 brain samples, we grouped the nuclei into five major cell types: astrocytes (Astro), excitatory neurons (ExN), inhibitory neurons (InN), oligodendrocytes (Oligo), and oligodendrocyte precursor cells (OPC), while dropping endothelial (Endo) cells and microglia (Micro) because of low fractions. Henceforth, we shall call these the Velmeshev data. We aggregated the expression of nuclei from each ASD sample to generate pseudo-bulk data for which we know the ground truth. We estimated the prior distribution using the snRNA-seq data from control samples. After analyzing the generated pseudo-bulk data to estimate CTS expression, we calculated the correlation between the true and estimated CTS expression, per cell type, per gene. Note that by generating the bulk data from the ASD samples and prior distribution from the control samples, we assessed the robustness of the method to utilizing distinct data sources for the analysis.

The correlation per gene based on bMIND was higher than those from TCA (Fig. 2B) and CIBERSORTx (Supplemental Fig. S1A) across cell types. Furthermore, because bMIND is a Bayesian approach, we evaluated the sensitivity of the estimates to the prior distribution specification by comparing bMIND to bMIND_rp (Fig. 2B) and observed that results from bMIND were relatively accurate even when the prior was not. Nonetheless, a precise prior can improve sample-level CTS estimates; for instance, the correlation between estimated expression and the truth across genes, for each cell type and each sample, was considerably higher using bMIND (Fig. 2C). Finally, to show that bMIND has the ability to incorporate additional measures of bulk expression from the same samples, we conducted realistic simulations and showed that more measures increased the estimation accuracy (Supplemental Fig. S1B). The bulk data were simulated with measured cell type fractions and sample-level CTS expression derived from snRNA-seq data (Velmeshev et al. 2019). To show that bMIND works for other tissue types, we repeat the simulations with a single-cell data set from heart (Litviňuková et al. 2020). With the generated pseudo-bulk data, bMIND's CTS estimates were more accurate than TCA (Supplemental Fig. S1C,D).

### bMIND has good power in CTS differential analysis

To evaluate the performance of bMIND for CTS DE analysis, we conducted extensive simulation studies, assessing its false discovery rate (FDR) and power. Using these simulated data, we planned to compare performance of bMIND to csSAM (Shen-Orr et al. 2010); however, csSAM had zero power in these settings. Other options for CTS DE analysis were TCA (Rahmani et al. 2019) and CIBERSORTx (Newman et al. 2019). As reported in the literature (Jing et al. 2019), however, we observed inflated FDR using TCA (Supplemental Fig. S1E) and thus did not consider it for CTS DE analysis. CIBERSORTx (Newman et al. 2019) was not open source and thus was not suitable for extensive simulation studies. In contrast, CellDMC (Zheng et al. 2018) was suitable and its performance relative to bMIND was evaluated in our simulations.

**Figure 3.**
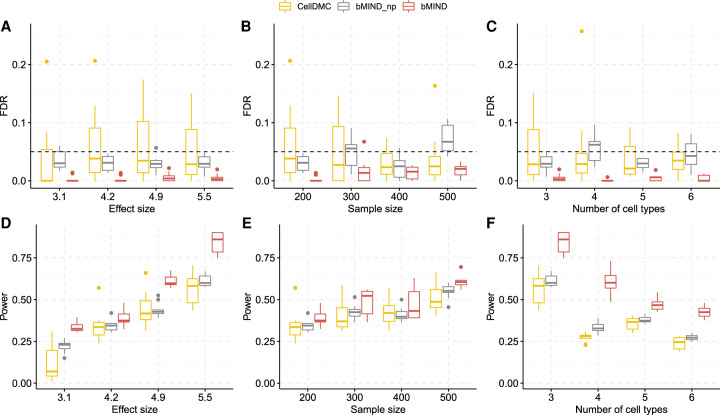
FDR and power simulation with true cell type fractions. (*A*–*C*) FDR as a function of effect size in DEGs (*A*), the sample size (*B*), and the number of cell types (*C*). (*D*–*F*) Power as a function of effect size (*D*) in DEGs, the sample size (*E*), and the number of cell types (*F*). bMIND_np represents a version of bMIND with non-informative prior. bMIND uses true mean hyperparameters but large variance hyperparameters (10^3^× true variances). If not specified, the total sample size is 200 and number of cell types is three. The average effect size is 4.2 for *B* and *E* and 5.9 for *C* and *F*. All simulation scenarios are replicated 10 times.

We assessed FDR and power as a function of effect size, the number of cell types, and sample size. Under all simulation scenarios, bMIND controlled FDR (Fig. 3A–C) at the nominal level of 0.05. bMIND (with informative prior) had improved power as compared to bMIND_np (with non-informative prior), which had greater power than CellDMC (Fig. 3D–F; Zheng et al. 2018). As expected, the power of CTS DE analysis increased with the effect size differentiating cases and controls (Fig. 3D) and with the number of samples evaluated (Fig. 3E), but it decreased as the number of cell types estimated increased (Fig. 3F). When we repeated the simulations with noisy and estimated cell type fractions, no inflation of the FDR was observed (Supplemental Fig. S2).

Next, to evaluate CTS DE in data for which we have an independent estimate of truth, we compared bMIND with results obtained from fluorescence-activated cell sorting (FACS) data for Alzheimer's disease (AD). Srinivasan et al. (2020) collected 113 FACS samples from human AD cases and controls, including 25 microglia samples, and 27 endothelial samples. From this experiment, they identified 66 DE genes in microglia and 135 in endothelial cells. For comparison to their results, we analyzed 85 AD and 99 control bulk samples from Brodmann area 36 (Wang et al. 2018) using bMIND, with a non-informative prior, to assess DE for more than 5000 genes expressed in brain cells (Grubman et al. 2019). We observed a strong correlation in log fold change between FACS-sorted cell data and bMIND for CTS DEGs for microglia and endothelial cells (Supplemental Fig. S3; Srinivasan et al. 2020). Thus, we conclude bMIND can successfully infer individual and CTS gene expression from bulk data to reveal meaningful CTS DE signal in the data.

### CTS differential expression analysis of ASD brain tissue

**Figure 4.**
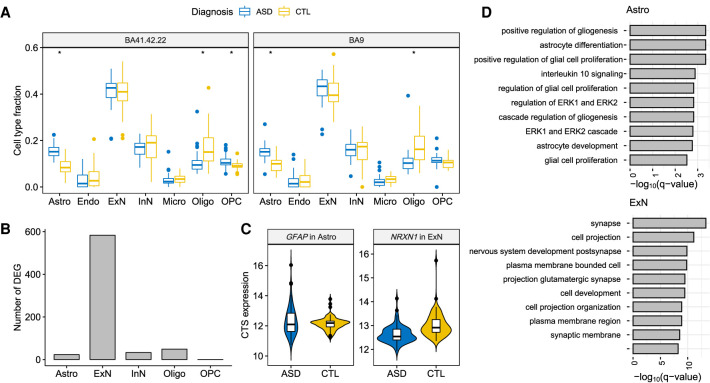
CTS differential expression analysis of autism. (*A*) Estimated cell type fractions for two cortical regions of the PsychENCODE UCLA-ASD data (Parikshak et al. 2016). (*) Significance after Bonferroni adjustment (*P*-value <0.05/14) comparing fractions of ASD and control samples. (*B*) Number of CTS DEGs identified by bMIND in each cell type. (*C*) Examples of bMIND-identified CTS DEGs. (*D*) Gene Ontology enrichment analysis for CTS DEGs in astrocytes and excitatory neurons: top 10 terms with FDR <0.05.

The snRNA-seq Velmeshev data provide a resource for analyzing bulk RNA-seq data related to autism in two ways: first, the data can be used for a prior for bMIND; and second, because the nuclei were drawn from ASD cases and controls, they can be used directly to assess DEGs. With these data as reference, we analyzed the PsychENCODE UCLA-ASD bulk RNA-seq cortex data, also obtained from brain tissue samples from subjects diagnosed with ASD and from control subjects (Parikshak et al. 2016). First, we estimated cell type fractions using Bisque (Jew et al. 2020) and then inferred CTS using bMIND. Similar to the findings based on snRNA-seq data (Velmeshev et al. 2019), we found that there were more astrocytes and fewer oligodendrocytes in ASD than control samples (Fig. 4A). Because microglia and endothelial cells showed average fractions below 0.05 (Velmeshev et al. 2019), we dropped these cell types before differential expression analysis. Using bMIND we identified 688 CTS DEGs over five major cell types (FDR <0.05 and absolute log_2_ fold change >0.14) (Supplemental Table S1). For comparison, analysis of the 41 snRNA-seq samples produced 513 CTS DEGs (Velmeshev et al. 2019) with the same criteria. Most of the CTS DEGs identified by bMIND were from excitatory neurons (Fig. 4B), which concurs with the snRNA-seq findings (Velmeshev et al. 2019). In contrast, CellDMC (Zheng et al. 2018) identified 5631 DEGs in inhibitory neurons and 2502 DEGs in excitatory neurons (FDR <0.05). Because CellDMC has been shown to detect too many signals in analyses of real data (Rahmani et al. 2019), our results suggest that the method is not robust to violations in the model assumptions and thus highly variable in its results.

When comparing CTS DEGs detected by bMIND and snRNA-seq data (Velmeshev et al. 2019), and examining significant results found in excitatory neurons, we obtained 33 genes in common (Fisher's exact test *P*-value = 4.3 × 10^−19^), including *NRXN1* (Fig. 4C). We also discovered some CTS DEGs using bMIND that had not previously been identified by Velmeshev et al. (2019), for instance, astrocyte marker gene *GFAP* (Fig. 4C). Among the bMIND-identified CTS DEGs, six genes (*GFAP*, *NRXN1*, *LRRC4C*, *KCNMA1*, *RORB*, *SLC6A1*) were among the 102 ASD risk genes discovered by Satterstrom et al. (2020) (Fisher's exact test *P*-value = 0.04) and 49 genes were among the SFARI autism gene list (Abrahams et al. 2013) (Fisher's exact test *P*-value = 2.5 × 10^−9^). As compared with the top 50 marker genes for each cell type derived from snRNA-seq data (Velmeshev et al. 2019), there was a significant enrichment of CTS DEGs as markers in astrocytes and excitatory neurons (Fisher's exact test *P*-value = 8.3 × 10^−4^ and 7.8 × 10^−8^, respectively).

We then evaluated the CTS DEG sets with Gene Ontology (GO) enrichment analysis (Fig. 4D; Supplemental Table S2; Raudvere et al. 2019). CTS DEGs identified in astrocytes were significantly enriched in the regulation of gliogenesis, astrocyte differentiation/development, and glial cell proliferation. Correspondingly, the CTS DEGs in excitatory neurons were enriched in glutamatergic (excitatory) synapse and nervous system development. We further parsed this set of enriched terms using REVIGO (Supek et al. 2011), a tool for clustering and interpreting long lists of GO terms. ASD DEGs were associated with 488 enriched GO terms. REVIGO identified two key themes for these terms, cell projection organization and neurotransmitter transport, as well as more minor themes of nervous system development and regulation of GTPase activity.

### CTS differential expression analysis of brain tissue from Alzheimer's disease subjects

In the second case study, we conducted CTS DE analysis for Alzheimer's disease. We analyzed bulk RNA-seq data from brain samples of subjects diagnosed with AD and unaffected control subjects from three projects: Mayo Clinic RNA-seq data (Allen et al. 2016), Mount Sinai Brain Bank (MSBB) (Wang et al. 2018), and Religious Order Study and the Memory and Aging Project (ROSMAP) (Bennett et al. 2018). We used AD snRNA-seq data (Mathys et al. 2019) for reference and the Bisque algorithm (Jew et al. 2020) to estimate cell type fractions. Following the cell clustering in the snRNA-seq data (Mathys et al. 2019), we focused on six cell types: Astro, ExN, InN, Micro, Oligo, and OPC.

**Figure 5.**
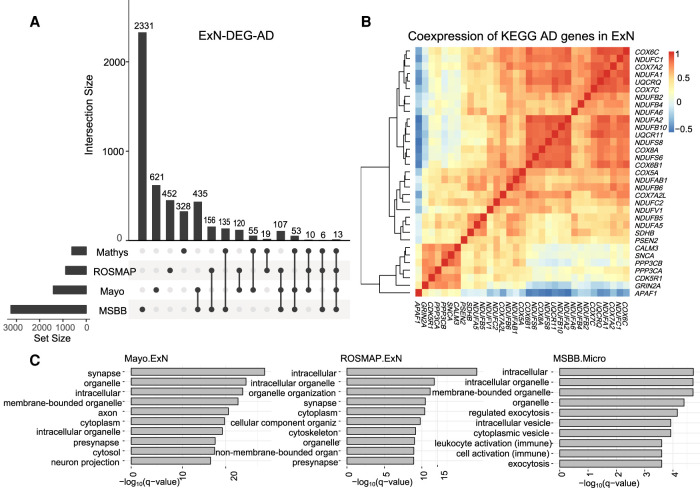
CTS differential expression analysis of Alzheimer's disease. (*A*) Intersection of DEGs in excitatory neurons (ExN DEGs) related to AD identified in snRNA-seq data (Mathys et al. 2019) and three bulk RNA-seq data sets (ROSMAP, Mayo, and MSBB) by bMIND. (*B*) Correlation matrix of the intersection of ExN DEGs in Mayo data and the KEGG AD gene pathway. Correlation is computed using bMIND estimated sample-level CTS expression. (*C*) Gene Ontology enrichment analysis for ExN DEGs for Mayo and ROSMAP bulk data and microglia DEGs for MSBB data (MSBB.Micro). Here, we present the top 10 terms with FDR <0.05 for each cell type.

Using bMIND, we estimated sample-level CTS expression and detected CTS DEGs related to AD with FDR <0.05 (Supplemental Table S1). Similar to the findings based on snRNA-seq of AD (Mathys et al. 2019), most identified CTS DEGs were from excitatory neurons, a finding that comports with the observed selective vulnerability of excitatory neurons in the brain of AD samples (Leng et al. 2021). We compared the ExN DEGs identified by the snRNA-seq study (Mathys et al. 2019) and bMIND from the three bulk data sets (Fig. 5A). The different numbers of DEGs can be explained by sample size and brain region heterogeneity. At the bulk expression level, an existing study (Marques-Coelho et al. 2021) also found more DEGs in the temporal lobe (Mayo data and MSBB Brodmann areas 22 and 36) than in frontal lobe (ROSMAP data and MSBB Brodmann areas 10 and 44). When we contrasted the ExN DEGs found from snRNA-seq (Mathys et al. 2019), we observed significant overlap with bMIND ExN DEGs for both the Mayo and MSBB data (Fisher's exact test *P*-value = 3.9 × 10^−13^ and 1.8 × 10^−5^, respectively).

CTS DEGs in excitatory neurons identified by the Mayo data were enriched in KEGG Alzheimer's disease pathway (Kanehisa and Goto 2000) (Fisher's exact test *P*-value =1.5 × 10^−5^). To illustrate how genes worked together in Alzheimer's disease at the cell type level, we took advantage of bMIND's estimates of sample-level CTS expression to construct a coexpression network of a subset of genes expressed in excitatory neurons (Fig. 5B); here, the genes illustrated were those shared by the two gene sets, ExN-DEG-AD and KEGG Alzheimer's disease pathway. We also conducted Gene Ontology enrichment analyses (Raudvere et al. 2019) for DEGs in different cell types (Supplemental Table S2). As expected, enriched terms for DEGs from excitatory neurons were enriched in synaptic and neuronal functions. For example, using REVIGO to assess the large number (683) of enriched GO terms for Mayo data identified several key themes: vesicle-mediated transport in the synapse, regulation of catabolism, and chemical synaptic transmission; and more minor themes of organelle organization and macro- and autophagy. DEGs in microglia, however, were enriched in immune processes (Fig. 5C).

### Contrasting functions of DEGs from excitatory neurons of ASD versus AD subjects

The bulk of DEGs for both ASD and AD arose from excitatory neurons, presenting an opportunity to learn more about both phenotypes based on commonalities and differences of enriched GO terms. (Here, we focused on DEGs from the temporal lobe of Mayo subjects.) ASD DEGs were enriched for 309 GO terms that were not shared with enriched terms for AD DEGs. From these terms, REVIGO identified one key theme, regulation of synapse organization, and two minor themes, amino acid transport and anatomical structure morphogenesis (Supplemental Fig. S4A). This was somewhat different than the key themes associated with the entire set of ASD DEGs, namely, cell projection organization and neurotransmitter transport. In fact, all these themes are likely important to liability for ASD (Satterstrom et al. 2020).

There were 504 enriched GO terms specific for AD DEGs, from which REVIGO identified key themes of vesicle-mediated transport and regulation of catabolism. More minor themes were organelle organization, macro- and autophagy, and protein/macromolecule modification (Supplemental Fig. S4B). These themes were quite similar to those identified from the entire list of enriched GO terms for AD DEGs.

Next, we asked how REVIGO interpreted the enriched terms that were shared between ASD and AD. Although 179 GO terms were identical, the FDR *Q*-values REVIGO used to prioritize them were not. On the contrary, there was no relationship between ASD and AD *Q*-values for these shared terms (*P*-value = 0.68, paired Wilcoxon test). The major themes that emerged for ASD were cell projection organization and neurotransmitter transport, quite similar to those for the entire set of enriched GO terms associated with ASD DEGs (Supplemental Fig. S4C). For AD, however, the major theme was regulation of cellular component organization, a theme that recurred across the different partitions of enriched GO terms for AD DEGs (Supplemental Fig. S4D).

### CTS eQTL analysis of GTEx V8 brain data

To generate a resource of inferred CTS eQTLs for various brain regions, we analyzed the latest GTEx brain data (The GTEx Consortium 2020) (V8) using bMIND. We first obtained the cell type fractions for each GTEx bulk sample via non-negative least squares and signature matrix derived from Darmanis et al. (2015) and described in Wang et al. (2020). We then estimated subject-level CTS expression for 11 GTEx brain regions, after combining replicates for frontal cortex and cerebellum: amygdala, cerebellum, anterior cingulate cortex, frontal cortex, hippocampus, hypothalamus, substantia nigra, caudate, nucleus accumbens, putamen, and spinal cord. For each region, gene expression was estimated for six cell types: Astro, Endo, ExN, InN, Micro, and Oligo. The summary statistics of significant (FDR < 0.05) gene-variant pairs were saved in GitHub folder (https://github.com/randel/bMIND_GTEx8_signif_region_CTS_eQTLs_cis).

**Figure 6.**
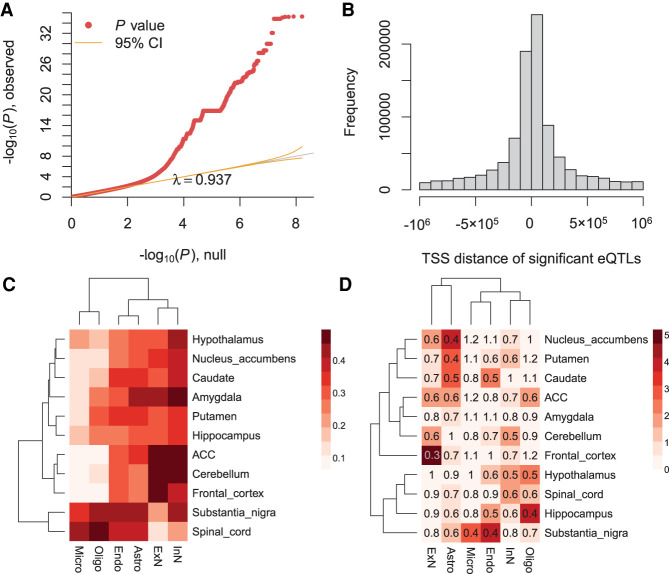
Region-specific CTS eQTL analysis with GTEx V8 brain data. (*A*) The QQ-plot of *P*-values from eQTL analysis. Here, we show an example of microglia in substantia nigra. (*B*) The enrichment of significant eQTLs near TSS. Here, we present an example of excitatory neurons in the frontal cortex. (*C*) Fraction of GTEx brain bulk eQTLs detected as CTS eQTLs in each brain region. (ACC) Anterior cingulate cortex. (*D*) The enrichment analysis of ASD genes in region-specific CTS eGenes (genes with eQTLs). The heatmap color denotes −log_10_ transformed Benjamini–Hochberg adjusted *P*-values (Benjamini and Hochberg 1995) based on two-sided Fisher's exact tests, and the number represents the odds ratio.

To evaluate the results of eQTL mapping, we first confirmed that the eQTL analysis *P*-values were well-calibrated (Fig. 6A; Supplemental Fig. S5) and eQTLs were enriched near the transcriptional start site (TSS), as expected (Fig. 6B; Supplemental Fig. S6). Next, we hypothesized that many of our region-specific CTS eQTLs would match the GTEx regional analysis of eQTLs using bulk data (The GTEx Consortium 2020). To make this comparison, we calculated the fraction of bulk eQTLs per region as detected as region-specific CTS eQTLs by bMIND (Fig. 6C), noting substantial concordance in general. In addition, as might be expected, the eQTL mapping fractions were highly correlated with the average cell type fraction per region, with a Pearson's correlation of 0.88. The high concordance reveals the important role of cell type abundance in bulk data analysis, and both analyses verify the replicability of our CTS analysis.

To assess the utility of bMIND's region-specific CTS eQTLs, we assessed the connection between ASD genes (Satterstrom et al. 2020) and genes with eQTLs (eGenes). Using pLI score (Lek et al. 2016) as a measure of gene conservation, we first replicated a previous finding that eQTLs do not tend to occur in very conserved genes (Lek et al. 2016; Werling et al. 2020). For instance, in ExN of frontal cortex, the odds ratio of being eGenes and conserved (pLI ≥ 0.995) was 0.53 (Fisher's exact test *P*-value =1.2 × 10^−31^). Because conserved genes were less likely to be eGenes and ASD genes tended to be very conserved (Satterstrom et al. 2020), it is reasonable to predict that ASD genes were less likely to be eGenes. This speculation was verified with enrichment analysis of ASD genes as eGenes. Curiously, relatively fewer ASD genes (Satterstrom et al. 2020) were likely to be eGenes in excitatory neurons of the frontal cortex (Fig. 6D), which thus far is the most related cell type and brain region associated with autism. In large part, this pattern emerges because ASD genes tend to have higher expression levels, as do cortical excitatory neurons, and ASD genes tend to be highly conserved.

## Discussion

We develop a Bayesian algorithm, bMIND, to provide CTS expression for bulk RNA-seq samples with prior information derived from scRNA-seq data. This approach addresses the limitation of bulk RNA-seq, namely, it arises as a convolution of CTS gene expression profiles, and the limitation of scRNA-seq data, namely, ample technical noise and a limited number of samples. Yet, bMIND builds on the unique advantages of each data type, which include a large sample size of bulk RNA-seq and CTS gene expression profiles from scRNA-seq. We conduct extensive simulations to compare bMIND with state-of-the-art methods, demonstrating that bMIND improves the estimation accuracy and differential testing power while controlling FDR. Through analysis of CTS differential expression for brain samples of subjects diagnosed with ASD or unaffected, as well as a similar design for Alzheimer's disease, we show the utility of bMIND to enhance the understanding of etiology with cell type resolution. Finally, by analysis of the latest GTEx V8 data using bMIND, we obtain CTS eQTLs for 11 brain regions. To the best of our knowledge, this is the most comprehensive brain CTS eQTL resource with which we verify existing findings, and we believe it will prove valuable for numerous studies.

When bMIND was used to determine CTS DEGs from ASD and control postmortem cortical tissue, most DEGs were identified in excitatory neurons (Fig. 4). This was also true of an earlier ASD study (Velmeshev et al. 2019), which used snRNA-seq data to obtain CTS DEGs directly from cortical excitatory neurons and other cell types. The DEGs from these studies share significantly more genes than expected by chance. Moreover, bMIND's CTS DEGs show significant overlap with the 102 genes implicated in ASD by a recent exome sequencing study (Satterstrom et al. 2020) and with a larger curated list of ASD genes (Abrahams et al. 2013). Finally, GO enrichment analysis of the bMIND CTS DEGs highlights exactly the processes ASD researchers have come to expect, namely, glutamatergic (excitatory) synapse, nervous system development, cell projection organization, and neurotransmitter transport. These are consistent themes from enrichment analysis of genes directly implicated in ASD by genetic studies (Voineagu et al. 2011; Parikshak et al. 2013; Willsey et al. 2013; De Rubeis et al. 2014; Gandal et al. 2018a; Polioudakis et al. 2019; Ruzzo et al. 2019; Satterstrom et al. 2020), with one important caveat. By necessity, individuals cannot be diagnosed with ASD at an early age. Thus, all of the postmortem bulk cortical data studied here come from subjects well past the fetal stage, during which rapid cell differentiation occurs. Thus, by design, we would not expect bMIND to capture DEGs from this critical stage of development. Nonetheless, bMIND's CTS DEGs identified from postnatal cortical tissue are consistent with genetic and neurobiological expectations for ASD, providing strong evidence for the validity of the individual-level CTS gene expression estimated by bMIND.

Perhaps because this cell type has higher expression levels and is thus better powered to detect DEGs, most CTS DEGs identified by bMIND from AD versus control postmortem brain tissue also derived from excitatory neurons (Fig. 5). These results paralleled results from unbiased snRNA-seq data sets contrasting AD and control cell types, all of which revealed excitatory neurons as a rich source of DEGs (Grubman et al. 2019; Mathys et al. 2019; Lau et al. 2020). Furthermore, bMIND's CTS DEGs showed significant overlap with a curated list of genes implicated in AD. Although bMIND's CTS DEGs showed enrichment in synaptic and neuronal functions, as it did for ASD, the patterns of enrichment were quite different from those for ASD. For AD, major themes included vesicle-mediated transport in the synapse, regulation of catabolism, and chemical synaptic transmission, whereas other themes included organelle organization and macro- and autophagy cellular processes. These processes have been implicated by various AD snRNA-seq studies of DEGs as well (Grubman et al. 2019; Mathys et al. 2019; Srinivasan et al. 2020; Marques-Coelho et al. 2021). bMIND also identified notable enrichment of CTS DEGs in astrocytes, highlighting gliogenesis, astrocyte differentiation/development, and glial cell proliferation. It did not, however, capture some important features of AD identified by snRNA-seq studies, including altered angiogenesis (Lau et al. 2020), age-differential microglial patterns of expression (Srinivasan et al. 2020), and role of oligodendrocytes in pathology (Mathys et al. 2019). With larger sample sizes and different brain regions sampled, we would expect that such features will emerge from future bMIND analyses of bulk gene expression. This raises an important point, however: bMIND is not a replacement for sc/snRNA-seq studies, it is a complement to them, providing another window into the processes underlying AD.

As described in Results, other methods are available for estimating CTS expression and differential testing, but bMIND is unique in that it provides a flexible tool, capable of seamlessly performing both estimation and testing while incorporating prior information. In comparison with TCA, our Bayesian estimation procedure obtains substantially more accurate estimates of CTS expression; our DE testing procedure is different from that in TCA; our mixed-effects model can naturally incorporate repeated measures of bulk expression; and the TCA model assumes that the CTS expression levels are independent across cell types, and our model allows correlation. Because it is a testing procedure, CellDMC differs more substantially: it can be only used for differential testing, but bMIND can also provide sample-level CTS expression for other downstream analyses; it only allows covariates that affect bulk data, but bMIND can additionally incorporate covariates that affect CTS expression; and it solely relies on bulk expression data, but bMIND can borrow prior information from sc/snRNA-seq studies.

In Wang et al. (2020), we introduced MIND to estimate CTS gene expression from bulk data by exploiting the correlation structure observed in multiple measures per subject. In contrast, bMIND incorporates prior information from sc/snRNA-seq data to circumvent the requirement of repeated measures, which are often not available. Moreover, bMIND can include covariates in the model, potentially removing the effects of confounding variables. Thus, harnessing existing information from both bulk and sc/snRNA-seq data, bMIND enhances the reproducibility of results across multiple technical platforms and studies. Indeed, in most settings, utilizing the prior information from sc/snRNA-seq data considerably improves the power and precision of CTS DE analysis, relative to MIND; however, when sc/snRNA-seq data are not available and many repeated measures have been sampled, MIND can obtain superior results because it does not use a non-informative prior and hence can more directly capitalize on the available information to obtain unbiased results.

Nonetheless, bMIND has limitations. For instance, bMIND provides more accurate CTS estimates for more abundant cell types. Thus, in those cell types the CTS differential expression analysis should be more powerful, all other things being equal. This issue also applies to the CTS differential expression analysis using cell-level data from scRNA/snRNA-seq, where cell types with more cells/nuclei quantified have much more power to detect CTS DEGs. More work will be needed to develop methods sensitive to less common cell types. In future work, we also plan a Bayesian approach to account for all sources of variation, such as the variation involving estimation of cell type fractions. Similar to other CTS analysis methods (Luo et al. 2020), bMIND is robust to moderate estimation error in cell type fractions. Here, we focus on RNA-seq data, but the approach for bMIND could also be used for the analysis of other omics data, such as DNA methylation. We will pursue this direction in future work.

Evaluation of bMIND is also limited by the resources currently available for validation. Consider, for example, our analyses of DEG between affected and unaffected subjects. If effect sizes for DEG were small, as suggested by findings from bulk data (Fromer et al. 2016; Gandal et al. 2018b), the number of subjects required to identify DEGs reliably is in the hundreds or thousands. For estimating DEGs from ASD and AD studies, however, cells were taken from 15 ASD and 16 unaffected subjects (Velmeshev et al. 2019) and from 24 AD and 24 unaffected subjects (Mathys et al. 2019), respectively. These studies are powered only to detect very large effect DEGs. Similar issues exist for sorted cell data. Consequently, our ability to validate bMIND's results in real data are hindered until snRNA-seq or sorted cell studies with larger sample sizes are available.

Note that CTS differential expression analysis is different from cell type enrichment analysis (Skene and Grant 2016). CTS differential expression analysis not only links diseases to specific cell types, but it also deepens the analysis to identify certain disease-related genes within those cell types. Although it remains a challenging task, we show the advantage and flexibility of estimating the virtual CTS expression profile for each bulk RNA-seq sample. In addition to the improved power to detect CTS DEGs and eQTLs, sample-level CTS expression enables the development of coexpression networks specific to certain cell types and other sample-level analyses.

## Methods

### Algorithm implementation

We implement the Bayesian mixed-effects models in bMIND with the MCMCglmm algorithm (Hadfield 2010), which fits a broad class of Bayesian generalized linear mixed-effects models based on MCMC approaches. It is flexible to incorporate normal prior for fixed effects and inverse-Wishart prior for the covariance matrix. As the conjugate prior, the inverse-Wishart prior facilitates estimation and allows the incorporation of the prior cell type covariance matrix estimated from scRNA-seq data explicitly. To make the computation feasible for all genes in the genome, we analyze one gene each time and run the analysis in parallel. To build a user-friendly software package, we integrate the following two steps: estimating cell type fraction and CTS expression. With users’ input of bulk data and either raw scRNA-seq data reference or signature matrix, we can output both cell type fractions via non-negative least squares or Bisque (Jew et al. 2020) and CTS expression via bMIND. When a phenotype is provided, the package will conduct CTS DE analysis and output *P*-values adjusted for multiple testing.

### Alternative CTS differential expression analysis

The output of bMIND is a three-dimensional array (gene × cell type × sample). With estimated sample-level CTS expression, we are able to conduct analyses that are previously only available using bulk RNA-seq data, deepening the analyses from tissue level to cell type level. Here, we focus on CTS differential expression (DE) analysis as an example. To control for false discovery rate (FDR) in CTS DE analysis, we propose a stringent testing procedure:

- We first conduct the multivariate analysis of variance (MANOVA) using CTS expression for each gene with respect to the phenotype of interest and claim a gene as a DEG by Benjamini–Hochberg adjusted *P*-values (Benjamini and Hochberg 1995).
- To find in which cell type a DEG is differentially expressed, we obtain CTS DE *P*-values by regressing the phenotype on CTS expression in that gene.
- A CTS DEG is determined if the CTS DE *P*-value is the minimal across cell types in a DEG and less than 0.05/*K*, where *K* is the number of cell types (Guo et al. 2010).

That is, we only detect the top significant signal across cell types within each gene. Existing snRNA-seq studies of ASD and Alzheimer's disease (Mathys et al. 2019; Velmeshev et al. 2019) support this testing scheme that most (79%–93%) CTS DEGs are only differentially expressed in a single cell type. The *P*-values are calculated with covariates adjusted.

### Simulation model of CTS DE testing

We first used estimated cell type fractions (***W***) for more than 600 samples from ROSMAP data. Then for each gene *j* = 1, …, 1000, we simulated CTS expression from $a_{j}^{0}∼\;N(0,0.01I)$ for controls, and simulated CTS expression from $a_{j}^{1}∼\;N(d,0.01I)$ for cases, where *d*_*jk*_ = *d* denotes the differential effect when gene *j* is a DEG in cell type *k*, and *d*_*jk*_ = 0 if not. There were 200*K*/3 gene-cell type pairs that were differentially expressed, where *K* is the number of cell types. The disease status ***y*** is simulated as binary with a probability of 0.5. With the error term (***e***_*j*_) and covariates (***c***^(1)^ and ***c***^(2)^) generated from standard normal distribution, we simulated the bulk expression for the *j*th gene as

$$x_{j}=I(y=0)\;*\;{Wa}_{j}^{0}+I(y=1)\;*\;{Wa}_{j}^{1}+c^{\left(1\right)}+Wb\;*\;c^{\left(2\right)}+e_{j},$$

where ***b*** is a vector of *K* × 1 with element *b*_*k*_ = 0.1*k* representing the covariate effect on the *k*th cell type. We explored multiple simulation scenarios by varying the sample size, number of cell types, and effect size, which is defined as *d*/*sd*(***X***), where ***X*** is the bulk expression.

### Data resources and analyses

To identify CTS genes expressed differently between ASD and unaffected (control) subjects, we analyzed bulk RNA-seq data from the PsychENCODE UCLA-ASD project (Parikshak et al. 2016), specifically 167 tissue samples from two cortical regions of 91 subjects (47 ASD and 44 control subjects). Subjects, who were mostly male (81%), ranged in age at death from 2 to 67, with 22 being the median age. For gene expression profiles specific to cell types, we used snRNA-seq data from an ASD study (Velmeshev et al. 2019), which collected 104,559 nuclei from 41 cortical samples taken from both ASD and control subjects. We also used these snRNA-seq data to generate realistic bulk data for the simulation studies. To evaluate the consistency of estimation results, we analyzed two similar bulk RNA-seq data sets (Parikshak et al. 2016; Velmeshev et al. 2019).

To identify CTS genes expressed differently between AD and control subjects, we used bulk RNA-seq data from three resources: the Mayo Clinic data (Allen et al. 2016) with 160 samples from the temporal cortex; Mount Sinai Brain Bank (MSBB) data (Wang et al. 2018) with 850 bulk samples from four cortical regions (Brodmann areas 10, 22, 36, and 44); and Religious Order Study and the Memory and Aging Project (ROSMAP) (Bennett et al. 2018) with 636 samples from the dorsolateral prefrontal cortex. We adopted a consistent definition for Alzheimer's disease (Braak score ≥4) across data sets. We compared our CTS DEGs to those from a snRNA-seq study (Mathys et al. 2019), which quantified the expression of 80,660 nuclei from the cortex of 48 subjects. All bulk RNA-seq and snRNA-seq data included both affected and control subjects.

Gene expression and genotype data from GTEx samples were obtained from the NCBI database of Genotypes and Phenotypes (dbGaP; https://www.ncbi.nlm.nih.gov/gap/) through accession number phs000424.v8.p2. After estimating subject-level region-specific CTS expression, we calculated *cis*-eQTLs for each brain region and cell type using MatrixEQTL (Shabalin 2012). To be included in this analysis, *cis* SNPs fell within ±1 Mb around each gene and had minor allele frequency >1%.

Sets of DEGs from both ASD and AD were analyzed for functional effects as determined by Gene Ontology (GO) enrichment analysis (Raudvere et al. 2019), using threshold FDR *Q* < 0.05. To capture the major functions of the DEGs obtained from ASD and from the Mayo study of AD, we analyzed their enriched GO terms by REVIGO (Supek et al. 2011), which assesses semantic similarity of GO terms (Schlicker et al. 2006), clusters similar terms, prioritizes more enriched terms for the semantic interpretation, and displays representative terms for the cluster. For these analyses, we used a similarity setting of 0.5, which favors shorter and semantically diverse lists of functions, as well as two default settings: semantic similarity measure SimRel and the database for GO term sizes “whole UniProt.” The terms were analyzed by the online version of REVIGO, which used GO release “go_monthly-termdb.obo-xml.gz” (January 2017) and UniProt-to-GO mapping file “goa_UniProt_gcrp.gaf.gz” (March 15, 2017).

### Software availability

The R software (R Core Team 2021) package is available as Supplemental Code and at GitHub (https://github.com/randel/MIND) with detailed bMIND tutorials.

## Supplementary Material

Supplemental Material
