# Supplementary material for: Bayesian estimation of cell type–specific gene expression with prior derived from single-cell data
Source: Genome Res. 2021 Oct;31(10):1807–18. doi: 10.1101/gr.268722.120 (PMC8494232; doi:10.1101/gr.268722.120)
Supplement: Supplemental Material [file supp_31_10_1807__DC1.html]

Bayesian estimation of cell type–specific gene expression with prior derived from single-cell data — Supplemental Material 

# Bayesian estimation of cell type–specific gene expression with prior derived from single-cell data

## Supplemental Material

- Supplemental\_Table\_S2.xlsx
- Supplemental\_Table\_S1.xlsx
- Supplemental\_Code.tar
- Supplemental\_Materials.pdf
