# Supplementary material for: Bayesian estimation of cell type–specific gene expression with prior derived from single-cell data
Source: Genome Res. 2021 Oct;31(10):1807–18. doi: 10.1101/gr.268722.120 (PMC8494232; doi:10.1101/gr.268722.120)
Supplement: Supplemental Material [file supp_gr.268722.120_Supplemental_Code.tar › MIND/MIND-manual.pdf]

Package ‘MIND’

November 12, 2020

Type Package

Title Using Tissue Expression to Estimate Sample-/Subject- And Cell-Type-Specific Gene Expression via Deconvolution

Version 0.3.1

Author Jiebiao Wang

Maintainer Jiebiao Wang <randel.wang@gmail.com>

Description
Methods to glean more insights from bulk gene expression: MIND and bMIND. MIND borrows information across multiple measurements of the same tissue per subject, such as multiple regions of the brain, using an empirical Bayes approach to estimate subject- and cell-type-specific (CTS) gene expression via deconvolution. The bMIND algorithm provides Bayesian estimates of sample-level CTS expression for each bulk sample.

Depends nnls, doParallel, foreach, MCMCglmm

License GPL

Encoding UTF-8

LazyData true

RoxygenNote 7.1.1

URL https://github.com/randel/MIND

BugReports https://github.com/randel/MIND/issues

R topics documented:

bMIND 2
est_frac 4
example 5
get_prior 5
mind 6

Index **7**

`bMIND` *The bMIND algorithm to estimate sample-level cell-type-specific expression and conduct CTS differential expression (DE) analysis*

Description

It calculates the Bayesian estimates of sample- and cell-type-specific (CTS) gene expression, via MCMC. For all input, dim names are recommended if applicable.

Usage

```
bMIND(
  bulk,
  frac = NULL,
  sample_id = NULL,
  ncore = NULL,
  profile = NULL,
  covariance = NULL,
  y = NULL,
  covariate = NULL,
  nu = 50,
  V_fe = NULL,
  nitt = 1300,
  burnin = 300,
  thin = 1,
  frac_method = NULL,
  sc_count = NULL,
  sc_meta = NULL,
  signature = NULL,
  signature_case = NULL,
  case_bulk = NULL
)
```

Arguments

`bulk`	bulk gene expression (gene x sample). We recommend log2-transformed data for better performance, except when using Bisque to estimate cell type fractions, raw count is expected for Bisque. If the max(bulk) > 50, bulk will be transformed to log2(count per million + 1) before running bMIND.
`frac`	sample-specific cell type fraction (sample x cell type). If not specified (NULL), it will be estimated by non-negative least squares (NNLS) by providing signature matrix or Bisque by providing single-cell reference.
`sample_id`	sample/subject ID vector. The default is that sample ID will be automatically provided for sample-level bMIND analysis, otherwise subject ID should be provided for subject-level bMIND analysis. Note that the subject ID will be sorted in the output and different sample_id would produce slightly different results in MCMCglmm.
`ncore`	number of cores to run in parallel for providing sample/subject-level CTS estimates. The default is all available cores.

profile	prior profile matrix (gene by cell type). Gene names should be in the same order of bulk, and cell type names should be in the same order as frac. If not specified (NULL), the bulk mean will be supplied.
covariance	prior covariance array (gene by cell type by cell type). Gene names should be in the same order of bulk, and cell type names should be in the same order as frac. If not specified (NULL), bulk variance / sum(colMeans(frac)^2) will be supplied.
y	binary (0-1) outcome/phenotype vector for CTS DE analysis. It can also be a factor with two levels. Should be the same length and order as sample_id or sort(unique(sample_id)) and row names of covariate.
covariate	matrix for covariates to be adjusted in CTS differential testing.
nu	hyper-parameter for the prior covariance matrix. The larger the nu, the higher the certainty about the information in covariance, and the more informative is the distribution. The default is 50.
V_fe	hyper-parameter for the covariance matrix of fixed-effects. The default is 0.5 * Identity matrix.
nitt	number of MCMC iterations.
burnin	burn-in iterations for MCMC.
thin	thinning interval for MCMC.
frac_method	method to be used for estimating cell type fractions, either 'NNLS' or 'Bisque'. **All arguments starting from this one will be used to estimate cell-type fractions only, if those fractions are not pre-estimated.**
sc_count	sc/snRNA-seq raw count as reference for Bisque to estimate cell type fractions.
sc_meta	meta data frame for sc/snRNA-seq reference. A binary (0-1) column of 'case' is expected to indicate case/control status.
signature	signature matrix for NNLS to estimate cell type fractions. Log2 transformation is recommended.
signature_case	signature matrix from case samples for NNLS to estimate cell type fractions. Log2 transformation is recommended. If this is provided, signature will be treated as signature matrix for unaffected controls.
case_bulk	case/control status vector for bulk data when using case/control reference to estimate the cell type fractions for case/control subjects separately.

Value

A list containing the output of the bMIND algorithm (some genes with error message in MCMCglmm will not be outputted, e.g., with constant expression)

A	the deconvolved cell-type-specific gene expression (gene x cell type x sample).
SE	the standard error of cell-type-specific gene expression (gene x cell type x sample).
Sigma_c	the covariance matrix for the deconvolved cell-type-specific expression (gene x cell type x cell type).
mu	the estimated profile matrix (gene x cell type).
frac	the estimated cell type fractions (sample x cell type).
pval	the p-values of CTS-DE testing (cell type x gene).
qval	the q-values of CTS-DE testing by MANOVA and BH FDR adjustment (cell type x gene).

References

Wang, Jiebiao, Kathryn Roeder, and Bernie Devlin. "Bayesian estimation of cell-type-specific gene expression per bulk sample with prior derived from single-cell data." bioRxiv (2020).

Examples

```
data(example)
bulk = t(na.omit(apply(example$X, 1, as.vector)))
frac = na.omit(apply(example$W, 3, as.vector))
colnames(bulk) = rownames(frac) = 1:nrow(frac)

# with provided cell type fractions
deconv1 = bMIND(bulk, frac = frac, y = rbinom(n = nrow(frac), size = 1, prob = 0.5),
    ncore = 2)

set.seed(1)
data(signature)
bulk = matrix(rnorm(300 * ncol(bulk), 10), ncol = ncol(bulk))
rownames(bulk) = rownames(signature)[1:nrow(bulk)]
colnames(bulk) = 1:ncol(bulk)

# without provided cell type fractions
deconv2 = bMIND(bulk, signature = signature[, -6], y = rbinom(n = nrow(frac), size = 1,
    prob = 0.5), ncore = 2)
```

---

est_frac *Estimating cell type fractions with a signature matrix using non-negative least squares (NNLS)*

Description

It calls the nnls package to estimate cell type fractions of bulk data using a pre-estimated signature matrix. It is recommended to keep the row and column names of the input data.

Usage

```
est_frac(sig, bulk)
```

Arguments

`sig`	signature matrix (marker gene x cell type).
`bulk`	bulk data that need to be deconvolved (gene x tissue sample).

Value

A matrix containing the estimated cell type fractions (tissue sample x cell type). Row sums have been normalized to be 1 per sample.

`example` *A data example*

Description

A data list for demonstration.

Value

A list containing

- `X` bulk gene expression (gene x subject x measure).
- `W` subject-specific cell type fraction (subject x measure x cell type).

Examples

```
data(example)
```

`get_prior` *get prior CTS profile and covariance matrix from single-cell data*

Description

It calculates prior CTS profile and covariance matrix from single-cell data. The output can serve as hyper-parameters in bMIND.

Usage

```
get_prior(sc, meta_sc)
```

Arguments

- `sc` single-cell count matrix, gene x cell.
- `meta_sc` data.frame for meta of cells (cell x features, including columns ‘sample‘ (sample ID), ‘cell_type‘).

Value

A list containing

- `profile` CTS profile matrix (gene x cell type), in log2(CPM + 1) scale.
- `covariance` CTS covariance matrix (gene x cell type x cell type).

References

Wang, Jiebiao, Kathryn Roeder, and Bernie Devlin. "Bayesian estimation of cell-type-specific gene expression per bulk sample with prior derived from single-cell data." bioRxiv (2020).

mind — *The Multi-measure INdividual Deconvolution (MIND) algorithm*

Description

It calculates the empirical Bayes estimates of subject- and cell-type-specific gene expression, via a computationally efficient EM algorithm.

Usage

```
mind(X, W, maxIter = 100, tol = 0.001, verbose = F, ncore = 4)
```

Arguments

`X`	bulk gene expression (gene x subject x measure).
`W`	subject-specific cell type fraction (subject x measure x cell type).
`maxIter`	maximum number of iterations for the EM algorithm.
`tol`	tolerance level of absolute relative change of the log-likelihood to stop the EM algorithm.
`verbose`	logical, to print the detailed information for each iteration: iter (the iteration number), logLike_change, sigma2_e, mean(diag(Sigma_c))).
`ncore`	number of cores to run in parallel

Value

A list containing the output of the EM deconvolution algorithm

`A`	the deconvolved cell-type-specific gene expression (gene x cell type x subject).
`mu`	the estimated profile matrix (gene x cell type).
`iter`	the number of iterations used in the EM algorithm.
`Sigma_c`	the covariance matrix for the deconvolved cell-type-specific expression (cell type x cell type).
`sigma2_e`	the error variance.
`loglike`	the log-likelihood for each EM iteration.
`var_A`	the posterior covariance matrix for A (vectorized covariance matrix by subject).

References

Wang, Jiebiao, Bernie Devlin, and Kathryn Roeder. "Using multiple measurements of tissue to estimate subject-and cell-type-specific gene expression." Bioinformatics 36.3 (2020): 782-788. https://doi.org/10.1093/bioinformatics/btz619

Examples

```
data(example)

deconv = mind(X = example$X, W = example$W, ncore = 2)
```

Index

bMIND, 2

est_frac, 4
example, 5

get_prior, 5

mind, 6
