# Supplementary material for: Bayesian estimation of cell type–specific gene expression with prior derived from single-cell data
Source: Genome Res. 2021 Oct;31(10):1807–18. doi: 10.1101/gr.268722.120 (PMC8494232; doi:10.1101/gr.268722.120)
Supplement: Supplemental Material [file supp_gr.268722.120_Supplemental_Materials.pdf]

Supplementary Materials for "Bayesian estimation of cell type-specific gene expression with prior derived from single-cell data"

Jiebiao Wang [1], Kathryn Roeder [2,3] and Bernie Devlin [4]

[1]Department of Biostatistics, University of Pittsburgh, Pittsburgh, PA 15261, USA
[2]Department of Statistics and Data Science, Carnegie Mellon University, Pittsburgh, PA 15213, USA
[3]Computational Biology Department, Carnegie Mellon University, Pittsburgh, PA 15213, USA
[4]Department of Psychiatry, University of Pittsburgh School of Medicine, Pittsburgh, PA 15213, USA

Supplementary Figures

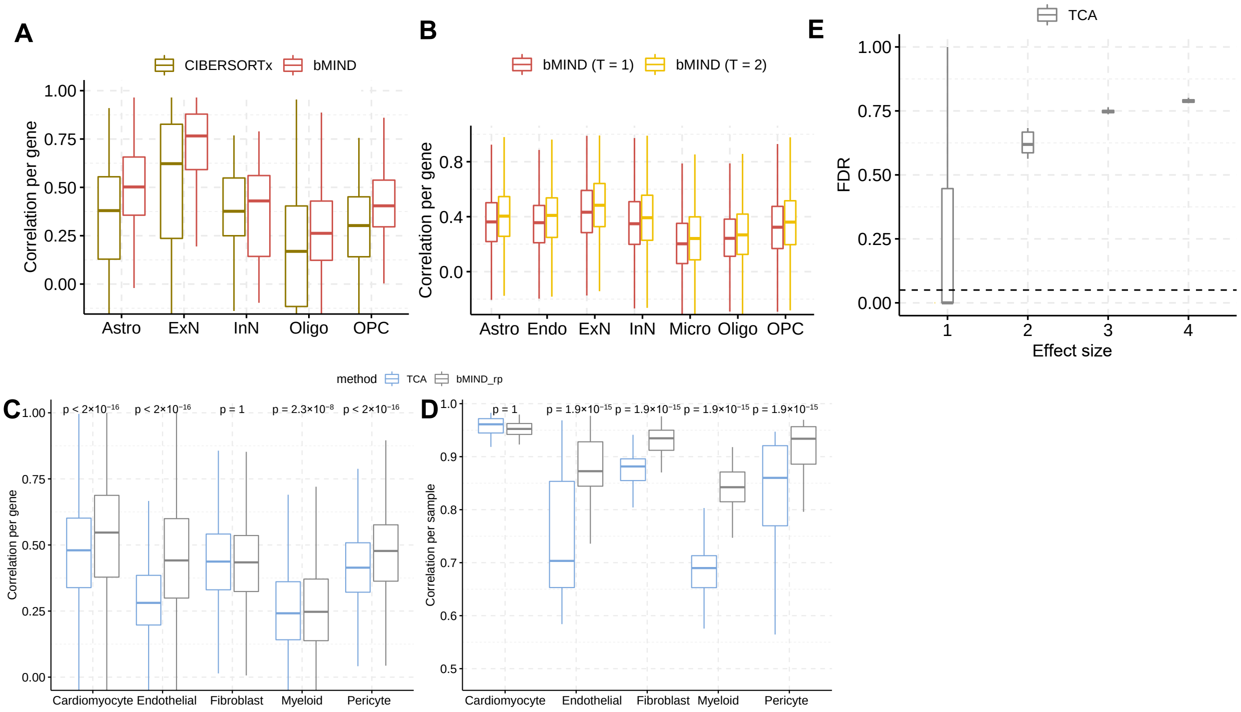

Supplementary Fig 1: *A*-*B*) Correlation comparison for estimated sample-level CTS expression. *A*) Analyzing pseudo-bulk data generated from snRNA-seq data (Velmeshev et al. 2019) of ASD cases. We compare correlation for each of 1000 genes and five cell types for CIBERSORTx and bMIND. The web version of CIBERSORTx for high-resolution/sample-level estimation only allows 1000 genes each run, and here it only outputs 3%-58% genes with varying estimates across samples. *B*) Comparing bMIND based on one and two measures. *C*-*D*) Analyzing pseudo-bulk data generated from single-cell heart data (Litviňuková et al. 2020). Correlation between estimated and true CTS expression per gene (*C*) and per sample (*D*). The p-values are obtained from paired one-tailed Wilcoxon test comparing TCA and bMIND_rp. *E*) FDR of TCA (Rahmani et al. 2019).

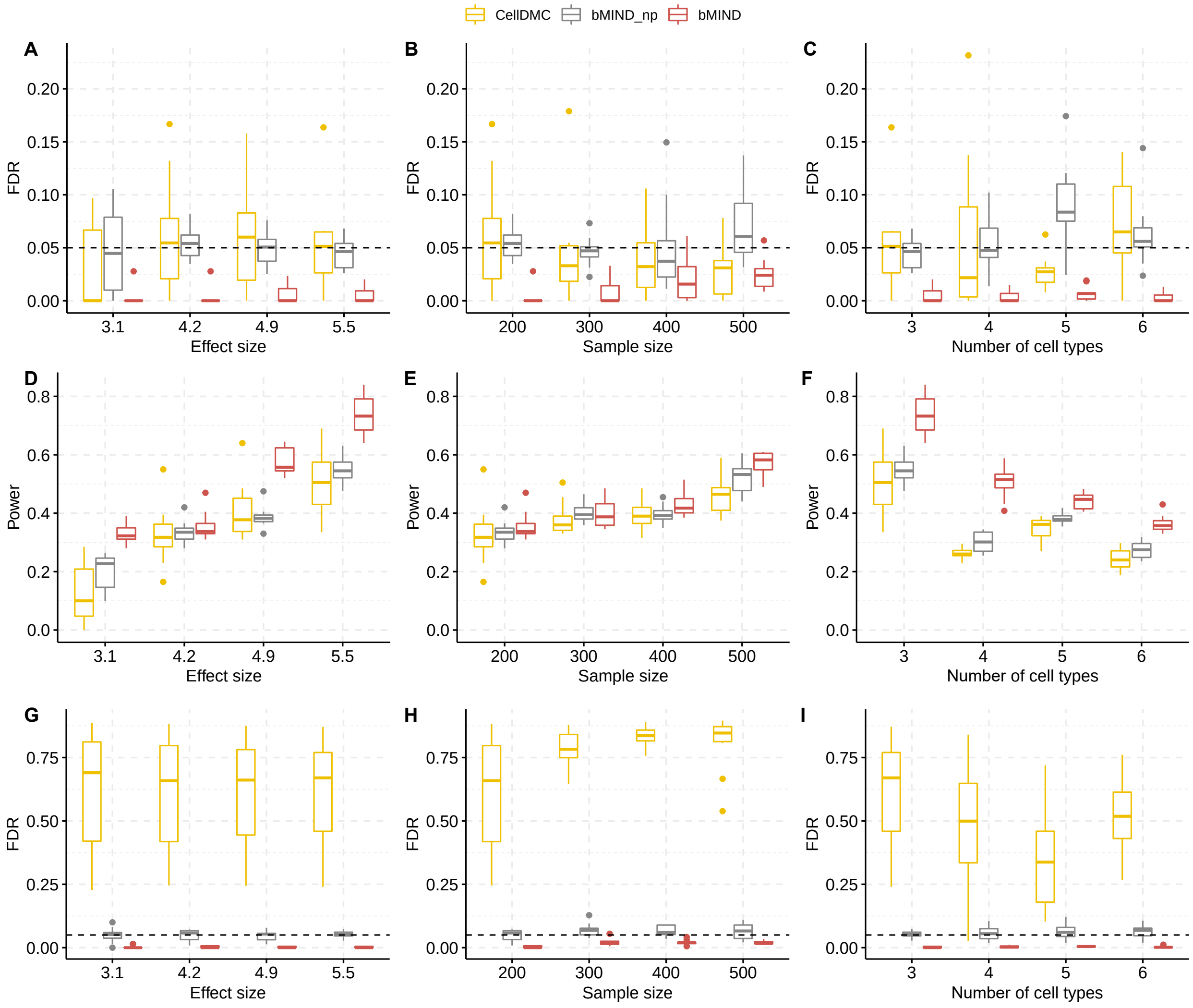

Supplementary Fig 2: FDR and power simulation with noisy cell type fractions. *A-C*) FDR as a function of effect size in DEGs (*A*), the sample size (*B*), and the number of cell types (*C*). *D-F*) Power as a function of effect size (*D*) in DEGs, the sample size (*E*), and the number of cell types (*F*). bMIND_np represents a version of bMIND with non-informative prior. bMIND uses true mean hyperparameters but large variance hyperparameters ($10^3\times$ true variances). If not specified, the total sample size is 200 and number of cell types is 3. The average effect size is 4.2 for *B,E*) and 5.9 for *C,F*). All simulation scenarios are replicated ten times. We randomly simulated noise from a uniform distribution between 0 and 0.08, adding noise to the true cell type fractions, and then normalized the total fraction to be one for each sample. This resulted in cell type fractions with an average MAE (mean absolute error) of 0.03 across simulation settings. *G-I*) FDR simulation with estimated cell type fractions. The cell type fractions are estimated using non-negative least squares. The average MAE of the estimated cell type fractions is 0.09.

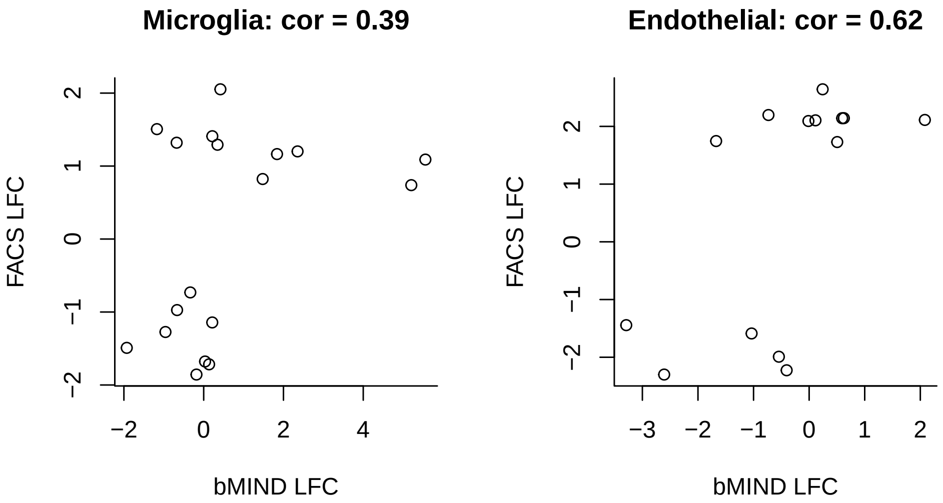

Supplementary Fig 3: LFC (log fold change) of FACS (fluorescence-activated cell sorting) sorted-cell data (Srinivasan et al. 2020) and bMIND deconvolved MSBB BM36 data for CTS-DEGs related to AD identified by Srinivasan et al. (2020). Given the small number of DEGs, the 95% confidence intervals for the correlation for bMIND in microglia and endothelial cells are (-0.09, 0.73) and (0.14, 0.87), respectively.

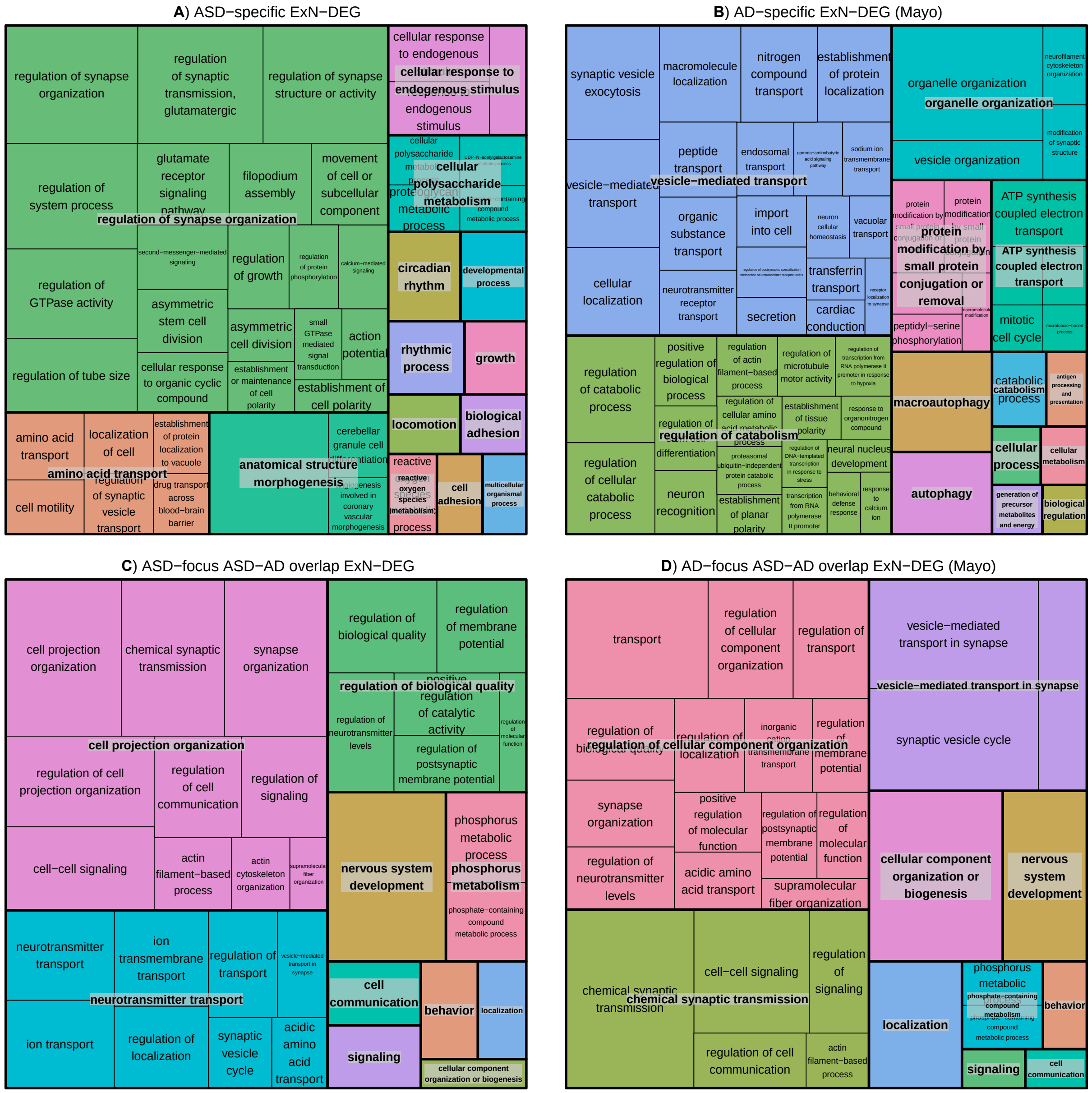

Supplementary Fig 4: REVIGO Gene Ontology treemap. *A*) ASD-specific ExN-DEGs, GO terms not presented in AD Mayo ExN-DEGs. *B*) AD-specific ExN-DEG in Mayo data, GO terms not presented in ASD-ExN-DEGs. *C*) Overlap GO terms of ASD-ExN-DEGs and AD-ExN-DEGs in Mayo data, using q-values from ASD. *D*) Overlap GO terms of ASD-ExN-DEGs and AD-ExN-DEGs in Mayo data, using q-values from AD.

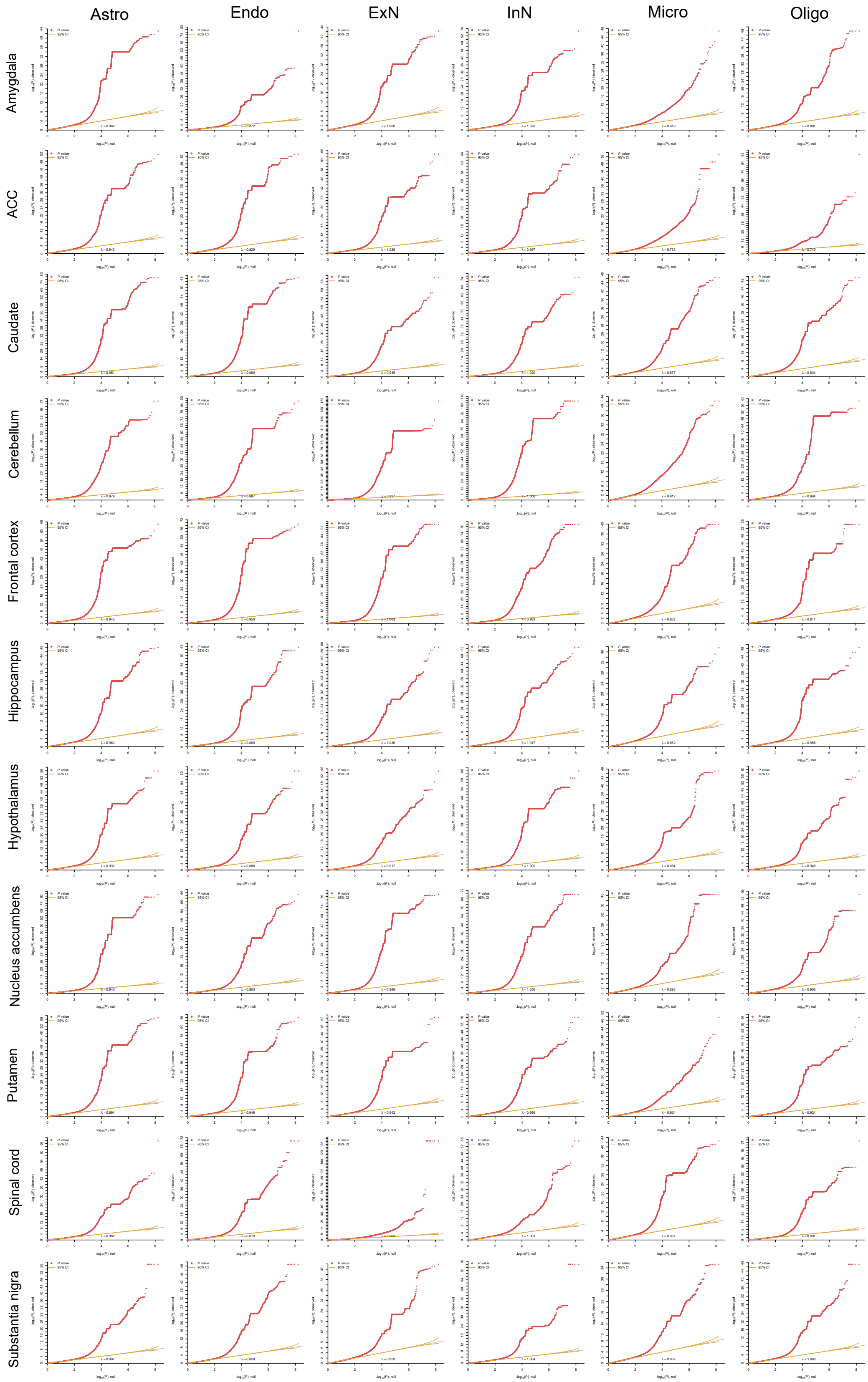

Supplementary Fig 5: The QQ-plot of p-values from eQTL analysis of each brain region (row) and each cell type (column).

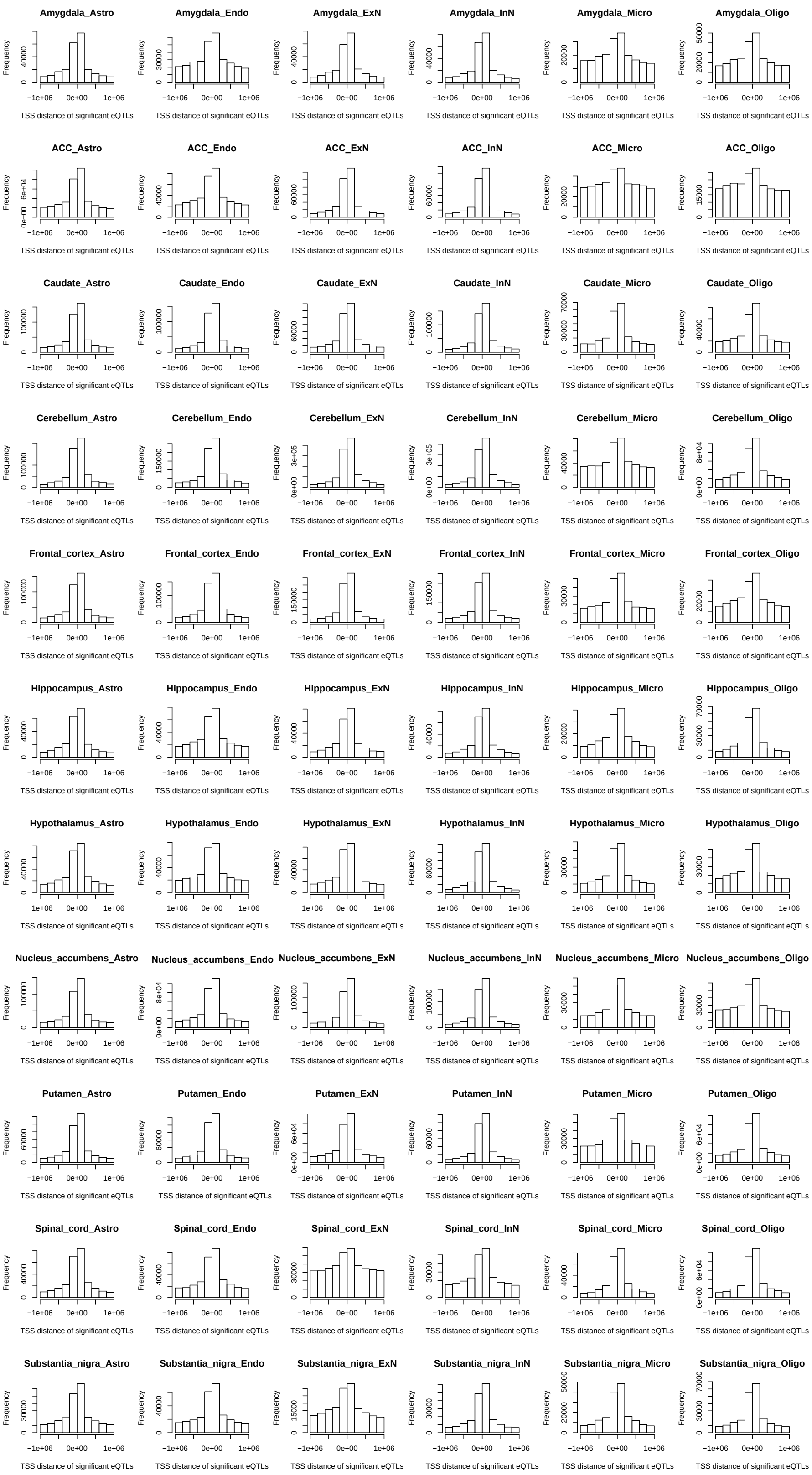

Supplementary Fig 6: The enrichment of significant eQTLs near transcriptional start site (TSS) for each brain region (row) and each cell type (column).

Supplementary Tables

Supplementary Table 1: CTS-DEG-ASD and CTS-DEG-AD identified by bMIND. The column names are gene, cell type, q-value, and log2 fold change (case - control).

Supplementary Table 2: Significant gene ontology enrichment results for CTS-DEG-ASD and CTS-DEG-AD identified by bMIND.

References

Litviňuková, M., Talavera-López, C., Maatz, H., Reichart, D., Worth, C. L., Lindberg, E. L., Kanda, M., Polanski, K., Heinig, M., Lee, M., *et al.*, 2020. Cells of the adult human heart. *Nature*, **588**(7838):466–472.

Rahmani, E., Schweiger, R., Rhead, B., Criswell, L. A., Barcellos, L. F., Eskin, E., Rosset, S., Sankararaman, S., and Halperin, E., 2019. Cell-type-specific resolution epigenetics without the need for cell sorting or single-cell biology. *Nature communications*, **10**(1):1–11.

Srinivasan, K., Friedman, B. A., Etxeberria, A., Huntley, M. A., van Der Brug, M. P., Foreman, O., Paw, J. S., Modrusan, Z., Beach, T. G., Serrano, G. E., *et al.*, 2020. Alzheimer's patient microglia exhibit enhanced aging and unique transcriptional activation. *Cell reports*, **31**(13):107843.

Velmeshev, D., Schirmer, L., Jung, D., Haeussler, M., Perez, Y., Mayer, S., Bhaduri, A., Goyal, N., Rowitch, D. H., and Kriegstein, A. R., *et al.*, 2019. Single-cell genomics identifies cell type–specific molecular changes in autism. *Science*, **364**(6441):685–689.
